# Optimize, Modulate, and Scale-up Resveratrol and Resveratrol Dimers Bioproduction in *Vitis labrusca* L. Cell Suspension from Flasks to 20 L Bioreactor

**DOI:** 10.3390/plants8120567

**Published:** 2019-12-04

**Authors:** Carole Lambert, Julien Lemaire, Hélène Auger, Arnaud Guilleret, Romain Reynaud, Christophe Clément, Eric Courot, Behnam Taidi

**Affiliations:** 1 Givaudan France SAS, Active Beauty, 51110 Pomacle, France; arnaud.guilleret@givaudan.com (A.G.); romain.reynaud@givaudan.com (R.R.); 2 LGPM, CentraleSupélec, Université Paris-Saclay, SFR Condorcet FR CNRS 3417, Centre Européen de Biotechnologie et de Bioéconomie (CEBB), 51110 Pomacle, France; julien.lemaire@centralesupelec.fr (J.L.); helene.auger@centralesupelec.fr (H.A.); behnam.taidi@centralesupelec.fr (B.T.); 3 Résistance Induite et Bioprotection des Plantes, EA 4707, SFR Condorcet FR CNRS 3417, UFR Sciences Exactes et Naturelles, Université de Reims Champagne-Ardenne, BP1039, 51687 Reims CEDEX 02, France; christophe.clement@univ-reims.fr (C.C.); eric.courot@univ-reims.fr (E.C.)

**Keywords:** plant cell culture, resveratrol, stilbene, viniferins, elicitation, methyl jasmonate, methyl-β-cyclodextrin, *Vitis labrusca*, bioreactors

## Abstract

Resveratrol and its oligomers are biologically active compounds. This work brings new insights for the bioproduction of *trans*-resveratrol with three dimers, pallidol, *trans*-ε-viniferin, and *trans*-δ-viniferin, in cell suspension of *Vitis labrusca*. Conditions of elicitation by methyl jasmonate were optimized for the production of stilbenes using statistical design of experiment. Bio-production of stilbenes was scaled-up to 5 L and in these conditions, *trans*-resveratrol concentrations reached 237 mg/L, and for pallidol 114 mg/L. The comparison of different elicitation modes (different elicitors, combination with cyclodextrins or adsorbent resin) allowed to reach particularly high concentrations of target molecules: Resveratrol 6.14 g/L, pallidol 0.90 g/L, δ-viniferin 0.54 g/L, and ε-viniferin 0.50 g/L. Scale-up to 20 L-stirring-bioreactor gave similar growth rates to those observed in shake flask culture, with a high production of resveratrol (4.23 g/L) and δ-viniferin (0.76 g/L). This work provides new strategies for the production of stilbenes in plant cell suspension for biological and commercial evaluation.

## 1. Introduction

Stilbenoids are plant metabolites derived from the phenylpropanoid pathway, particularly abundant in Vitaceae spp., of which approximatively 100 different compounds have been identified [1]. A great deal of interest has been exerted on the monomer *trans*-resveratrol because of its antioxidant properties and promising pharmacological activities in the fields of cancer treatment, inflammation reduction, atherosclerosis prevention, aging retardation, neurodegenerative disorder treatment, and combatting viral infections [2,3]. Resveratrol derivatives are attracting much more attention due to their chemical diversity and their higher biological activities than resveratrol itself [4,5,6,7]. For example, the dimer ε-viniferin demonstrated in vitro activities against bacteria [8], cancer cell lines [9,10], and β-amyloid aggregation [11], but also in vivo, it showed positive effects on the cardiovascular system [12]. Thus, the commercial demand for resveratrol and complex stilbenoids is ever-increasing.

Resveratrol is commercially available and is produced by extraction of *Fallopia japonica* roots or by biotechnology using transgenic micro-organisms [13]. Resveratrol oligomers are much rarer and could be retrieved, as well as resveratrol, from grapevine woody stems that contain a large variety of hydroxystlibenoids at concentrations up to 7 mg/kg DW (dry weight) [14,15]. Vine stems are a renewable material, but extraction of stilbenes requires organic solvents, and pesticides or heavy metals are susceptible to be retrieved in the final product.

Plant cell cultures are a useful production platform for natural compound synthesis and have even led to commercial use, especially in the cosmetic industry [16]. Despite the disadvantages presented by the slow growth rate of these cells, they do offer the possibility of producing complex compounds that otherwise are very challenging to obtain through chemical synthesis, or even in transgenic microorganisms. Dedifferentiated *Vitis* cell suspensions have been extensively studied for their ability to produce resveratrol and its glucoside piceid [13,17]. The use of inducers is required to obtain significant amounts of target metabolite [18]. Methyl jasmonate (MeJA) is a widely used elicitor and allows the production of 200–840 mg/L of intracellular *trans*-piceid and 200 mg/L of extracellular *trans*-resveratrol [19,20,21]. The extracellular secretion of resveratrol facilitates its recovery from the culture medium. The synergistic effect of methyl-β-cyclodextrins and MeJA has opened up new perspectives by inducing production of *trans*-resveratrol up to 3000 mg/L, and recent publications describe the intracellular signaling pathways involved [22,23,24]. Methyl-β-cyclodextrins act as elicitors, as well as cage molecules, protecting *trans*-resveratrol from oxidation [23,25]. More recently, in situ recovery of *trans*-resveratrol in cell culture medium by adsorbent resin was undertaken, and metabolite amounts produced reached 2700 mg/L [26,27]. These considerations highlight the fact that grapevine cells are suitable factories, at least for resveratrol production.

*Vitis* cells cultivated in vitro not only produce resveratrol and piceid, but a large diversity of compounds, from monomers like piceatannol, astringin, resveratroloside, resveratrol di- and tri- glucosides, to dimers such as pallidol, ε-viniferin, δ-viniferin, and leachianols F and G [4,20,28,29,30,31,32,33], and a new dimer named labruscol was recently identified in *V. labrusca* cell suspension [5]. Biological activities of resveratrol oligomers are attracting the attention of scientists, but their level of production in grapevine cell culture is low: In the order of milligrams per liter (mg/L) or milligrams per kilogram (mg/kg) of fresh weight [31,34,35]. So it is of great interest to increase their production.

The aim of the present work was to further the knowledge on the production of resveratrol and some of its oligomers by *Vitis* cells in bioreactor culture. The ability of a *Vitis labrusca* cell suspension to produce complex stilbenoids under improved conditions for elicitation with MeJA is reported. The production of complex stilbenoids was achieved in 5 L and 20 L fed-batch cultures. The effect of methyl-β-cyclodextrin combined with different elicitors and the use of an adsorbent resin were evaluated on resveratrol oligomers production.

## 2. Results

### 2.1. Optimization of MeJA Elicitation in Flask Experiments

One of the limiting factors in plant cell culture is the slow growth rate of cells, which increases the cost of production and extends the risk of contamination and equipment failure. It is then of importance to determine how to maximize the production of the desired compounds with the lowest cell concentration possible and to maximize the concentration of stilbenes in an elicited cell suspension. We supposed that the MeJA elicitation effect depended on the cell concentration and the sucrose content at the time of elicitation, as suggested by previous studies [35,36,37]. A full factorial experimental design was used to evaluate the combined effects of biomass, MeJA, and sucrose concentrations at the time of elicitation on the stilbene concentration. We chose to express the stilbene content in the medium in milligrams per gram of cells (dry weight, DW), named afterward production yield or yield. Indeed, relating metabolite content production to the biomass concentration is a way to highlight the conditions that allow higher production yield. This study was performed in shake flask cultures and the results were analyzed by stepwise regression, while taking into account primary effects, as well as bipartite and tripartite interactions. Preliminary assays (Figure S1) showed that major stilbenes reached their maximum 7 days after elicitation.

**Figure 1:**
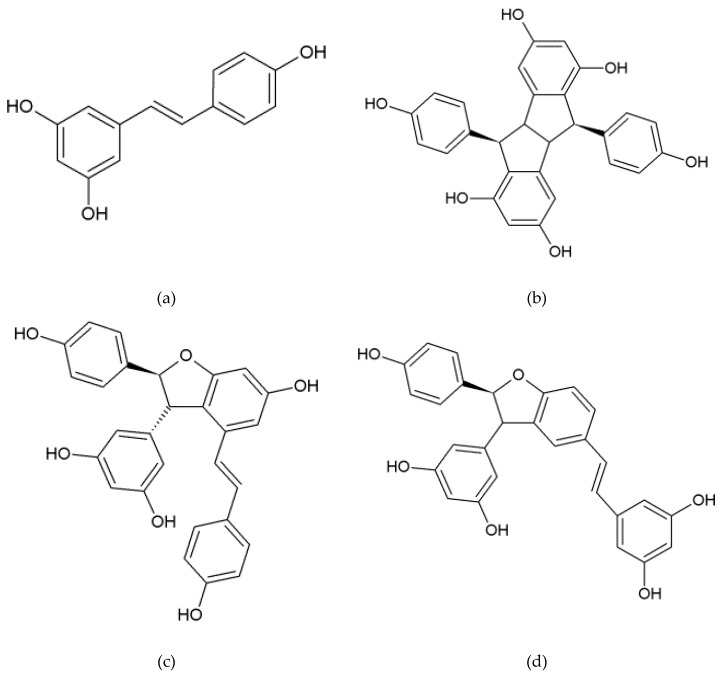
Chemical structure of stilbenes identified in the culture medium of elicited *Vitis labrusca* cell suspension. (**a**) *trans*-resveratrol, (**b**) pallidol, (**c**) ε-viniferin, (**d**) δ-viniferin.

We identified four stilbenes (Figure 1) in the culture medium of *V. labrusca* cells by LC–MS analysis: *Trans*-resveratrol ([M−H]^+^ 229, fragmented ion 135) and its dimers, pallidol ([M−H]^+^ 455, fragmented ion 361), δ-viniferin ([M−H]^+^ 455, fragment ions 437, 361) and the dimer ɛ-viniferin that was identified by comparison with pure standard. Pallidol is a bio-active compound and was recently demonstrated to regulate angiogenesis [38] and cancer-related signaling pathways [39]. These results are supported by a previous reported phytochemical study on *V. labrusca* cell suspension [4].

Statistical analysis was performed for the major compounds: Resveratrol and pallidol, based on their yield. In control cultures (not elicited), no stilbenes were detected in the medium after 7 days of incubation.

A statistical analysis (Student’s *t*-test) discriminated the significance of the three parameters (biomass concentration, sucrose content, and MeJA concentration) and their interactions (Table 1 and Table 2). Meanwhile, multiple regressions were performed to provide a function which estimates stilbene production from the normalized value of the three tested parameters (range between −1 and +1), respectively, for resveratrol and pallidol.

(*trans*-resveratrol mg/g DW) = 11.56 − 7.41B + 5.12M + 0.2S − 2.84BM + 4.97BS − 0.77MS − 2.91BMS (1)

(pallidol mg/g DW) = 11.71 − 5.14B + 0.50M + 1.75S + 0.40BM + 3.34BS − 2.19MS + 1.86BMS(2)

**Figure 2:**
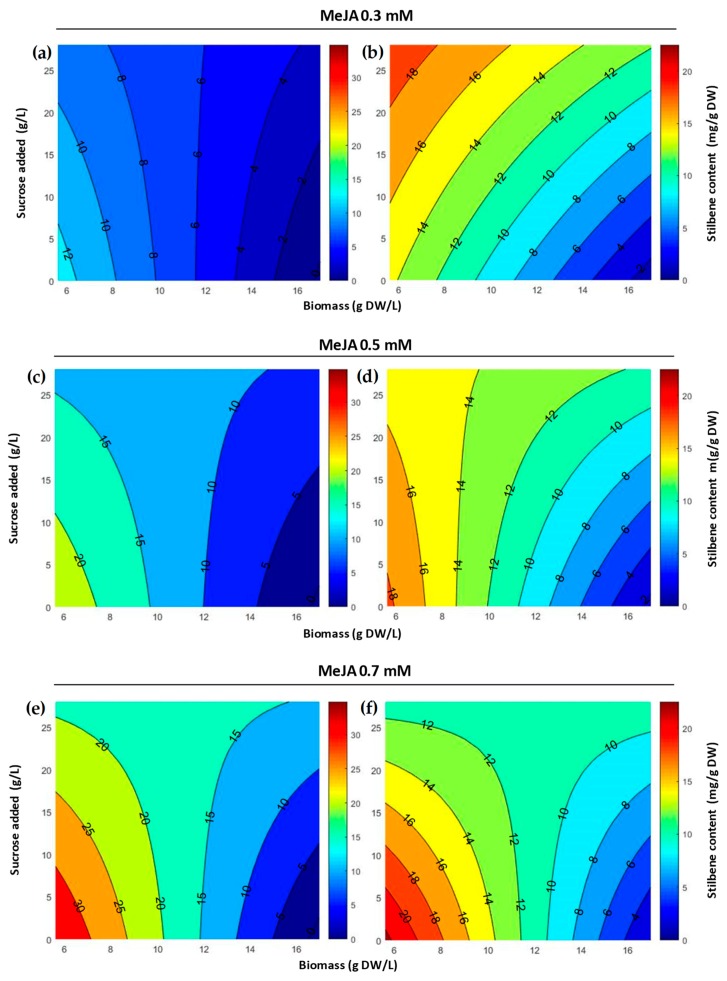
Surface response plot for resveratrol (**a**,**c**,**e**) and pallidol (**b**,**d**,**f**) production: Effect of methyl jasmonate (0.3, 0.5, and 0.7 mM), biomass (5.6, 10.5, and 17 g DW/L, dry weight per liter), and sucrose added (0, 14, and 28 g/L) concentrations after 7 days of elicitation. The color scales correspond to the level of production of resveratrol (0 (blue)–30 (red) mg/g cells DW) and pallidol (0 (blue)–20 (red) mg/g cells DW), respectively.

The response surface plots were generated to visualize the results (Figure 2). An analysis of variance (ANOVA) was also performed to assess the significance of the correlation: According to the F-value (28 for resveratrol and 43.1 for pallidol, with 22 degrees of freedom) and the corresponding *p*-value (<0.0001), both models were significant. Moreover, there was no significant lack of fit. Besides, the correlation coefficients (R^2^ = 0.86 for resveratrol, 0.91 for pallidol) indicate that 86 and 91% of the variability could be explained by the model for resveratrol and pallidol productions, respectively.

**Figure 3:**
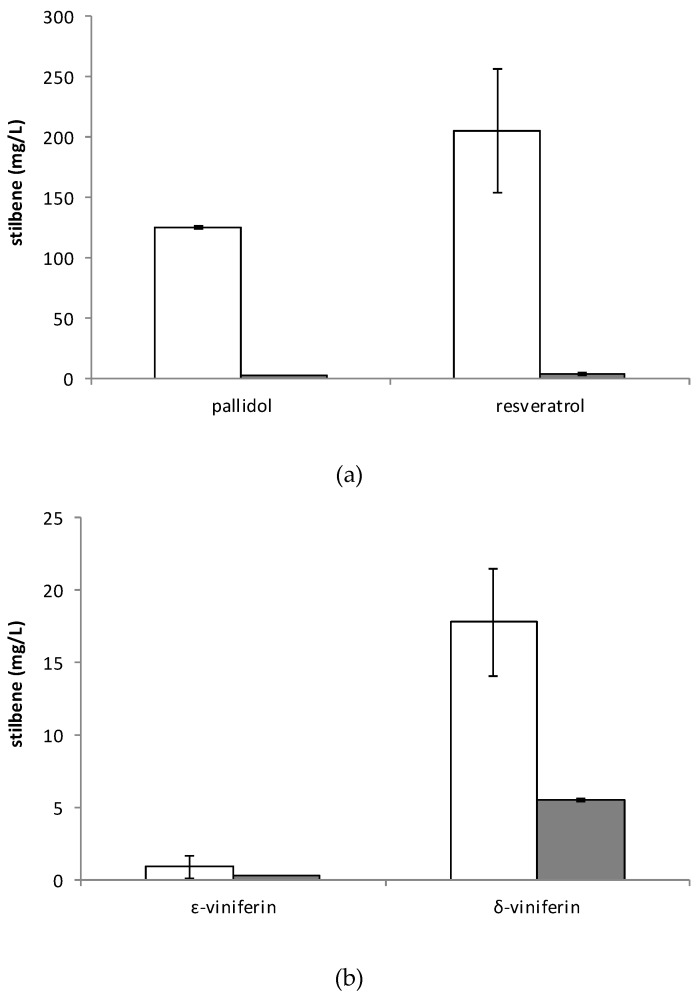
Concentration of (**a**) pallidol and *trans*-resveratrol and (**b**) ε-viniferin and δ-viniferin in *Vitis labrusca* cell suspension grown in shake flasks and elicited with methyl jasmonate 0.7 mM, 5.6 g DW cells/L and no sucrose supplementation (white bars, extracellular concentration; grey bars, intracellular concentration). Results are the mean of duplicate flasks and expressed in milligrams per liter (mg/L) of whole medium (extracellular medium and cells). The error bars represent the standard deviation.

For both resveratrol and pallidol concentrations, Figure 2e,f indicates that the optimal conditions (red zone) are: Biomass 5.6 DW g/L, MeJA 0.7 mM, and no sucrose supplementation. The maximal production of stilbenes in the culture medium (Figure 3) was: Resveratrol 36.7 mg/g DW (206 mg/L) and pallidol 22.3 mg/g DW (124.9 mg/L). We also quantified δ-viniferin 2.4 mg/g DW (17.8 mg/L) and ɛ-viniferin <1 mg/g DW (<5 mg/L).

As the highest stilbene production was reached with the lowest cell biomass tested, it suggests that lower cell concentrations could be evaluated. The observed resveratrol concentration was similar to that reported on *Vitis* 41B cell suspension subjected to MeJA elicitation: 209 mg/L [19]. According to Morales et al. [40], the maximal solubility of resveratrol in Gamborg medium is 214 mg/L, which could explain why the reported resveratrol concentrations, in medium not supplemented with solubilizer like methyl-β-cyclodextrins, did not exceed this value.

The quantification of intra- and extracellular stilbenes (Figure 3) confirmed that resveratrol and its derivatives are predominantly excreted in the culture medium: More than 98% of *trans*-resveratrol and pallidol, 76% of δ-viniferin, and 75% of ɛ-viniferin. In situ recovery of resveratrol in a culture medium of *Vitis* cells by an adsorbent resin has highlighted the interest of the extracellular localization of hydroxystilbenoids [27].

Our results indicate an intrinsic link between the three variables of biomass concentration at the moment of inoculation, MeJA, and sucrose concentration. For both resveratrol and pallidol, there was a strong bipartite interaction between biomass and sucrose concentrations [Equations (1) and (2)]. We observed (Figure 2) that, at the highest biomass level, the highest resveratrol yield was obtained at the highest sucrose concentrations. Belhadj et al. also described a positive effect of carbohydrate addition on resveratrol production [36]. Conversely, at the lowest biomass level, the sucrose concentration had a negative effect on the production of resveratrol. For given MeJA and sucrose concentrations, resveratrol and pallidol yields were always higher for low biomass conditions. Belchí-Navarro et al. also observed that in *Vitis vinifera* cv. Monastrell cells co-elicited with MeJA and methyl-β-cyclodextrin, the yield of resveratrol decreased as the initial biomass increased [22]. The authors also determined an optimal cell concentration at 5 g DW/L. They explained this by the ratio of the number of MeJA molecules and their receptors on the cell surface. The ratio MeJA concentration/biomass is a key element to understand resveratrol production, and the optimal ratio observed in this work was 0.125 mmol/g DW. Furthermore, it has been shown that the solubility of MeJA in the water liquid medium could be enhanced by its complexation with cyclodextrins, and then provoked the highest elicitation response [41].

### 2.2. Production of Stilbenes in 5 L Bioreactor under Optimized MeJA Elicitation

**Figure 4:**
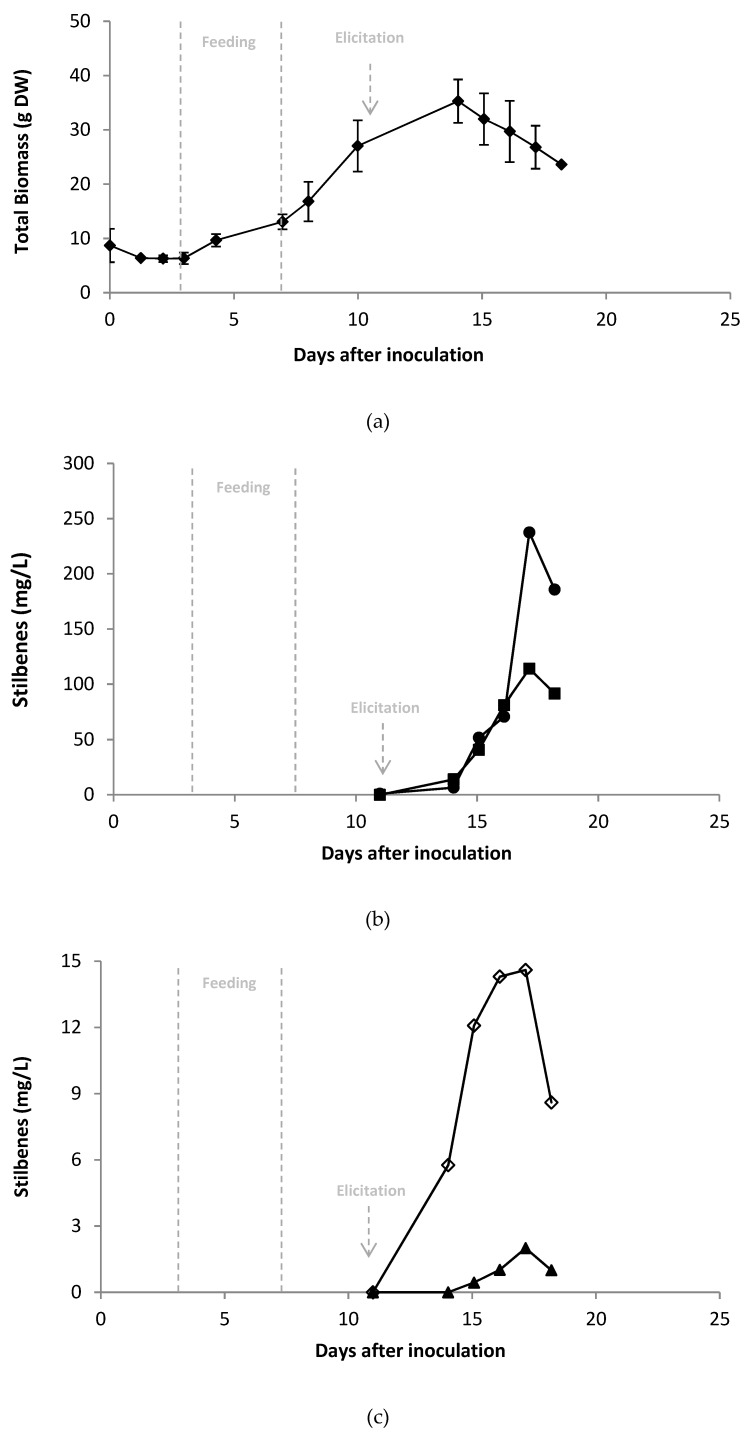
Growth curve (**a**) and time course of stilbene accumulation in the extracellular medium (**b**,**c**) of *Vitis labrusca* cells cultivated in a 5 L bioreactor elicited 11 days after inoculation by 0.7 mM methyl jasmonate (grey arrow). Day 0 corresponds to the inoculation of bioreactor with cells. Feeding indicates the period of time for which bioreactor was fed with Gamborg medium to allow biomass growth.

The optimal conditions determined by the factorial experiment were applied to a 5 L bioreactor culture: A biomass concentration at 5.6 g DW/L at the moment of elicitation, no sucrose supplementation, and a MeJA concentration of 0.7 mM (Figure 4a).

During the exponential phase, the doubling time of cells was 3.9 days. By comparison, doubling time in shake flask experiments was 4.6 ± 0.6 days (data not shown), so cultivation in the 5 L bioreactor did not modify the cells growth rate, although it is commonly accepted that shear stress negatively affects dedifferentiated plant cells. After elicitation, the biomass decreased, due to significant cell death caused by the MeJA elicitation. Stilbenes were quantified in the culture medium, from the time of elicitation (11 days after inoculation; Figure 4b,c) till the end of the culture.

The concentration of stilbenes peaked from 16 to 18 days (i.e., 5 to 7 days after elicitation) of culture, with δ-viniferin the first compound to accumulate. At this time point, the concentrations of resveratrol and its derivatives in the medium were similar to those in shake flask cultures: *Trans*-resveratrol 238 mg/L, followed by pallidol 114 mg/L, δ-viniferin 15 mg/L and ε-viniferin 2 mg/L. The resveratrol concentration in *V. labrusca* cell suspension medium, elicited by MeJA only, is high compared to *V. vinifera* cell suspensions that usually produce high content of *trans*-piceid, a resveratrol glucoside [18]. Such resveratrol concentration, under MeJA elicitation alone, was only reached in *Vitis* 41B, a cross-breeding of *Vitis Berlandieri* that also produces stilbene dimers [19]. It is interesting to observe that cell cultures developed from North American species like *V.labrusca* and *V. Berlandieri*, known to exert specific mechanism of resistance [42], produce and excrete high amounts of resveratrol. Moreover, to the best knowledge of the authors, this is the first report of such high pallidol concentrations in a cell suspension.

From literature and our own experiments we conclude that obtaining higher concentrations of stilbenes in the reaction medium is also related to other factors than biomass, carbohydrate content and elicitor concentration. Indeed stilbenoids solubility, their cell toxicity and degradability limit their bioproduction in plant cell suspensions and that led us to test ways to stabilize them in situ.

### 2.3. Stilbene Profile in Cell Suspension in the Presence or Absence of Adsorbent Resin or Methyl Beta-Cyclodextrins and Other Elicitors

In situ product recovery from the growth medium is common in biotechnological production of high-value bacterial compounds, and can be achieved with resins. Yue et al. demonstrated that HP2MGL, a non-aromatic resin, is highly effective for the recovery of resveratrol [27]. The use of methyl-β-cyclodextrins is a way to solubilize and stabilize stilbenes, as they form inclusion complexes with resveratrol and also act as elicitor [25,43]. Both strategies lead to very high resveratrol concentration in *V. vinifera* cell suspensions [13].

Few reports describe the impact of elicitors on resveratrol dimers, and we studied the impact of MeJA/resin and MeJA/methyl-β-cyclodextrins on stilbene profiles compared with simple MeJA treatment [34,44]. Flasks with 100 mL cell suspension were elicited with 0.5 mM MeJA, 0.5 mM MeJA plus 5 g of XAD1600N resin, and 0.5 mM MeJA plus 50 mM methyl-β-cyclodextrins. XAD1600N was selected among ten other adsorbent resins for its affinity to resveratrol and its derivatives (Figure S2).

**Figure 5:**
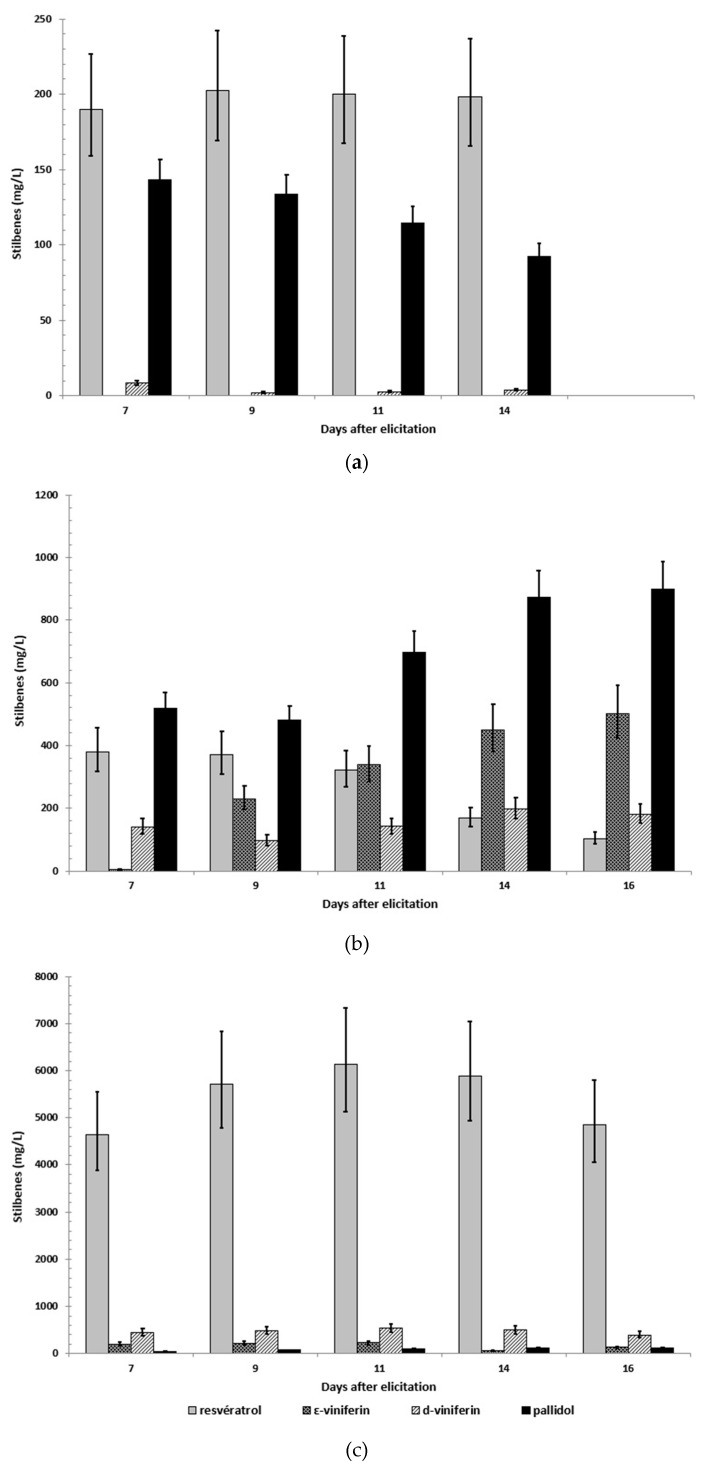
Time course of stilbene (*trans*-resveratrol, ε-viniferin, δ-viniferin, and pallidol) accumulation in the extracellular medium of *Vitis labrusca* cell suspensions treated with 0.5 mM methyl jasmonate (**a**), 0.5 mM methyl jasmonate and adsorbent resin XAD 1600N (**b**), and 0.5 mM methyl jasmonate and 50 mM methyl-β-cyclodextrins (**c**). Results are the mean of duplicate flasks. In the case of cells incubated with resins, stilbene content represents the sum of free and resin-bound extracellular compounds per liter of extracellular medium.

The accumulation of resveratrol and its dimers from day 7 to 16 after elicitation was followed in the extracellular medium (Figure 5). A specific extraction procedure was developed to retrieve molecules adsorbed on resin sot that the content of stilbenoids include, as well as free and resin-bound compounds. Figure 5a presents stilbene concentration for cells elicited with MeJA 0.5 mM. As previously observed, resveratrol and pallidol were the major components between days 7 and 14, with highest concentrations at 202.6 and 143 mg/L, respectively.

**Figure 6:**
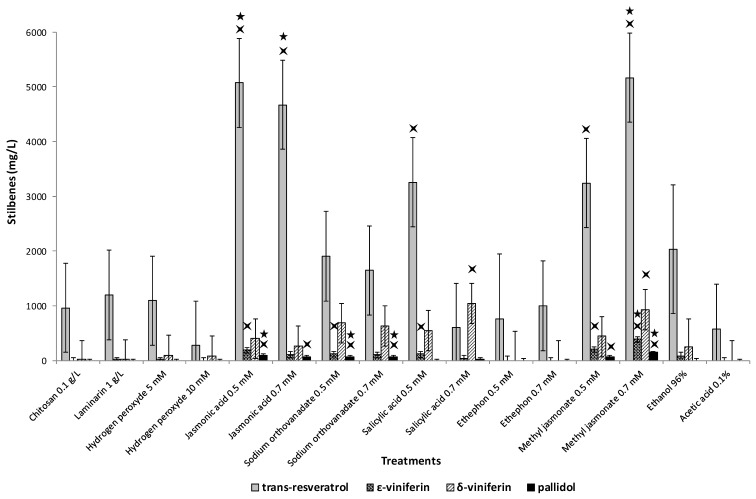
Stilbene accumulation (*trans*-resveratrol, ε-viniferin, δ-viniferin, pallidol) in the extracellular medium of *Vitis labrusca* cell suspension treated with 50 mM methyl-β-cyclodextrins supplemented with 0.1 g/L of chitosan, 1 g/L of laminarin, 5 or 10 mM of hydrogen peroxide, 0.5 or 0.7 mM of jasmonic acid, 0.5 or 0.7 mM of sodium orthovanadate, 0.5 or 0.7 mM of salicylic acid, 0.5 or 0.7 mM of ethephon, or 0.5 or 0.7 mM of methyl jasmonate. Ethanol 96% and acid acetic 0.1% were controls as these solvents served as carrier. The bar graph corresponds to mean concentration versus treatments performed in duplicate. Error bars were estimated from Tukey’s HSD (honestly significant difference) after performing an ANOVA, which demonstrated that means were significantly different (with 5% alpha risk). Four-pointed and five-pointed stars were placed above means significantly different from control “Acetic Acid 0.1%” and control “Ethanol 96%”, respectively.

Figure 5b presents stilbene concentration for flasks elicited with 0.5 mM in the presence of 5 g of adsorbent resin. More than 97% of total stilbenes was adsorbed on the resin (data not shown). Very different stilbene repartition was observed compared to Figure 6. Indeed, the major compound was pallidol, while it was resveratrol in all other experiments. Pallidol reached its maximum concentration at 16 days: 900.5 mg/L. The dimer ε-viniferin followed the same development and reached 500.2 mg/L at day 16, while δ-viniferin varied between 97.6 to 198.3 mg/L. Resveratrol decreased from day 7 (380.9 mg/L) to 16 (103.8 mg/L).

Figure 5c presents stilbene concentration for flasks elicited with 0.5 mM in the presence of 50 mM methyl-β-cyclodextrins. Again, the stilbene profile was very different from the two previous assays. Resveratrol was predominant from day 7 to 16 and reached 6141 mg/L. Dimers accumulated to a maximum concentration of 537.6 mg/L for δ-viniferin, 230.9 mg/L for ε-viniferin, and 116.0 mg/L for pallidol.

To our knowledge, it is the first time that such dimer concentrations are reported in grapevine cell culture, especially in the presence of adsorbent resin: Comparing the pallidol concentration in the extracellular medium, 900.5 mg/L, with the recently reported measure in the canes of *V. vinifera* cv. Merlot [45], 110 mg/kg, it appears that our production system, due to the concentration of pallidol and its lower chemical complexity, would be an interesting starting point for the purification of this active resveratrol dimer. The use of adsorbent resin also allowed a complete shift from resveratrol to dimers with a specific increase in pallidol and ε-viniferin: This is a very different profile compared to resveratrol and δ-viniferin accumulation with methyl-β-cyclodextrin-treated cells. It is difficult to propose a hypothesis to explain such differences, because the synthesis of stilbene dimers is still not fully elucidated, even though peroxidases are supposed to be responsible for the oxidative dimerization of resveratrol [46]. Nevertheless, these results point out the biotechnological interest of cyclodextrins and adsorbent resins to modulate the stilbene profile and guide the production of targeted dimers.

Figure 6 compares the impact of different elicitors, ever reported by the literature to stimulate resveratrol synthesis by grapevine cell culture, in combination with cyclodextrin. Jasmonic acid and MeJA were the most efficient elicitors of *V. labrusca* cells, followed by salicylic acid 0.5 mM. Salicylic acid 0.7 mM induced the highest concentration of δ-viniferin in the medium. The type of elicitor and its concentration is also a way to modulate the stilbene profile of *V. labrusca* cell suspension.

Based on our observations, we decided to scale-up the MeJA/methyl-β-cyclodextrins co-elicitation from 100 mL culture volume to 20 L.

### 2.4. Scale-up of A Co-Elicitation Methyl-Jasmonate/Methyl-β-Cyclodextrins Culture in 20 L Bioreactor

**Figure 7:**
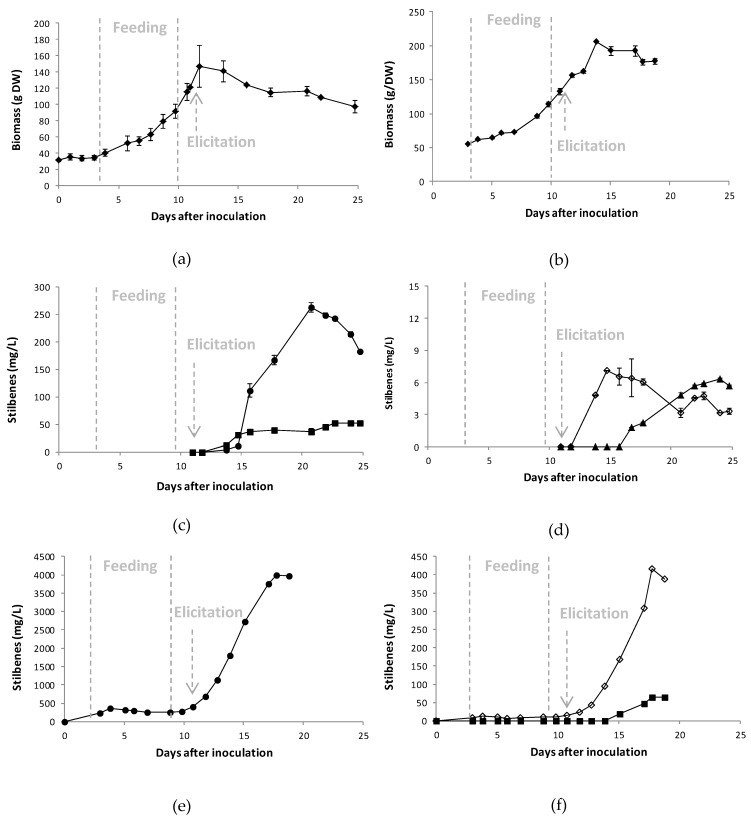
Growth curves (**a**,**b**) and time course of stilbene accumulation (**c**–**f**) in the extracellular medium of *Vitis labrusca* cell grown in a 20 L bioreactor and elicited by methyl jasmonate 0.5 mM and (**a**,**c**,**d**) or co-elicited with methyl jasmonate 0.5 mM and methyl-β-cyclodextrins 50 mM (**b**,**e**,**f**). For (**c**–**f**), *trans*-resveratrol (●), pallidol (■), δ-viniferin (◊), ε-viniferin (▲). Day 0 is the day of bioreactor inoculation with cells. Feeding indicates the period of time for which the bioreactor was fed with Gamborg medium to allow biomass growth. Methyl jasmonate treatment occurred after 11 days after inoculation in all experiments and is indicated by a grey arrow.

Co-elicitation with MeJA and methyl-β-cyclodextrins has been identified as an efficient treatment for resveratrol production [23], and in this study, we provided data on the effect of cyclodextrins on resveratrol oligomer synthesis (Figure 6). A co-elicitation MeJA/methyl-β-cyclodextrins was compared with a simple MeJA elicitation and scaled-up in 30 L bioreactor with 20 L working volume. Methyl-β-cyclodextrins were added to the medium at 50 mM and from the outset of the culture, as described by Belchí-Navarro et al. [22]. The growth curves in the absence/presence of cyclodextrins are presented in Figure 7a,b, respectively, and the time course of stilbene accumulation in Figure 7c–f.

In the medium not supplemented with methyl-β-cyclodextrins, the doubling time of cells was 4.6 days, and in the same order as those observed in the 5 L bioreactor (3.9 days) and shake flasks (4.6 days). Similar growth rates were observed by Vera-Urbina et al., in a 2 L stirred tank bioreactor [47]. The 20 L bioreactor was equipped with three 6-blade Rushton impellers and four baffles that increase turbulence, but also shear stresses compared to the 5 L bioreactor equipped with one 3-blades marine impeller and no baffles. Nevertheless, the growth of *V. labrusca* cells was effective in these conditions. Elicitation was performed 11 days after inoculation and the MeJA concentration used was 0.5 mM. As observed in the 5 L bioreactor, the first compound to reach its maximal concentration was δ-viniferin (7.1 mg/L) 14.7 days after elicitation (Figure 7c,d). This has been reported by Santamaria et al. [44]. Other stilbenes reached their concentration peaks between 20 and 23 days after elicitation: Resveratrol 263.3 mg/L, pallidol 53.3 mg/L, and ɛ-viniferin 5.9 mg/L. By comparing the results of both 5 and 20 L reactors in the absence of cyclodextrins, the production of stilbenes seemed to occur earlier in the 5 L reactor. This could be attributed to the difference in MeJA concentrations, as demonstrated by Donnez et al. [19], who found that the MeJA concentration significantly impacts the kinetics of resveratrol production: The lower the concentration of elicitor, the later the peak of resveratrol production.

In the presence of methyl-β-cyclodextrins, the doubling time of the cells was 6 days (Figure 7b), which was longer than those observed in shake flask culture, 5 L bioreactor, and 20 L cultures in the absence of cyclodextrins (4.6 days). Since methyl-β-cyclodextrins themselves elicited resveratrol production, stilbenes were quantified from the inoculation of the bioreactor. Before the addition of MeJA (0.5 mM) on day 11, the resveratrol concentration reached 367 mg/L and δ-viniferin was the only additional stilbene detected in the culture medium (16 mg/L). After co-elicitation, the resveratrol content increased very significantly, reaching 4000 mg/L (15-fold compared to MeJA elicitation). These results confirm the levels of resveratrol (4963 g/L) observed by us and reported by Bru and Pedreno in 80 mL culture [48]. The level of the dimer ε-viniferin increased 28-fold to 417 mg/L. Pallidol reached 66 mg/L, similar to that observed in 20 L bioreactor culture of cells elicited with MeJA alone (53.3 mg/L). The dimer ɛ-viniferin was not detected in the culture medium.

To our knowledge, this is the only study that has reported the scale up of resveratrol oligomers by grapevine cell culture to a such volume in stirred tank bioreactors. We demonstrated that the culture of *V. labrusca* cell suspension is possible in bioreactors inducing shear stress with an interesting production time of 18 days.

## 3. Materials and Methods

### 3.1. Cell Culture Maintenance

Cultures of *V. labrusca* (L.) cv. concord were established as described previously [49], and maintained at 23 °C in the dark in 300 mL Erlenmeyer flasks containing 100 mL of cell suspension on an orbital shaker (110 rpm, 2.5 cm deflection). The maintenance medium was Gamborg B5, containing vitamins (Duchefa Biokemie, Haarlem, The Netherlands) and supplemented with 30 g/L sucrose, 0.2 mg/L of kinetin, and 0.1 mg/L of naphthalene acetic acid [50]. Cell cultures were subcultured in shake flasks every week by inoculating the cells at 1/3 (v/v) into fresh medium.

### 3.2. Statistical Design of Experiment

To examine the combined effect of three parameters, a full factorial design 23 = 8 points with an additional center point, leading to a total of 9 experiments, was performed in triplicates. The variables were the dry biomass (5.6 (−1), 10.5 (0), and 17 (1) g DW/L), the MeJA concentration (0.3 mM (−1), 0.5 mM (0), and 0.7 mM (1)), and additional sucrose supplemented at the time of elicitation (0 (−1), 14 (0), and 28 (1) g/L) to a medium already containing 20 g/L of carbohydrate. The responses measured were the concentrations (in milligrams per gram of cells DW) of the major compounds produced 7 days after elicitation: *Trans*-resveratrol and pallidol. The experiment was designed and the results treated using the Matlab software (MathWorks, Natick, MA, USA).

For these experiments, the cells were first grown in a 5 L bioreactor operated in fed-batch mode (Biostat Bplus Sartorius, Göttingen, Germany) equipped with 3-bladed segment stirrers. Vessel geometry was H/D 2:1. The agitation rate was 100 rpm. The aeration rate was 0.15 vvm throughout the incubation period, obtained by adjusting the air flow rate to the culture volume on a daily basis. The bioreactor containing initially 2.5 L of Gamborg medium was inoculated with 500 mL of a 7-day-old shake flask culture at approximately 15 g DW/L. After 3 days, the feeding of the medium was started at 20 mL/h and was maintained until day 7, when the culture volume reached 4.5 L. The culture containing 10.5 g DW/L was harvested 10 days after inoculation and was used to prepare three batches of cells of different biomass concentrations: 5.6 g DW/L (150 g/L FW, fresh weight), 10.5 g DW/L (300 g/L FW), and 17 g DW/L (450 g/L FW). To obtain the required biomass concentration in the subsequent shake flask experiments, an adequate part of the cell suspension from the bioreactor was aseptically dripped through a stainless filter (250 µm mesh). If necessary, the medium thus recovered was used to dilute the cell suspension, while the cells (filter retentate) could be used to enrich the cell suspension without modification of the culture–medium composition.

The cell suspensions of different biomass concentrations were dispatched into flasks (100 mL/flasks) and 3 days after inoculation, MeJA was added. giving final concentrations of either 0.3, 0.5, or 0.7 mM. Similarly, additional sucrose at either 14 or 28 g/L was added to the flasks, in addition to any carbohydrate already present in the culture, as necessary, according to the experimental design. After 7 days of elicitation, stilbene contents were determined and expressed in milligrams per gram of cells (dry weight).

### 3.3. Treatment with MeJA Combined with Adsorbent Resin or Methyl-β-Cyclodextrins

Seven-day-old flask cell suspensions were diluted in Gamborg medium to obtain fresh cultures at 5.6 g DW/L (150 g/L FW). Three days after inoculation, cells were treated. One third of the flasks were elicited with ethanolic MeJA solution to reach a 0.5 mM final concentration and were considered as control condition. One third of the flasks were elicited with MeJA 0.5 mM in the same conditions, and a sterile nylon bag containing 5 g of adsorbent resin XAD 1600N (Dow Chemical, Midland, TX, USA) was introduced into each flask. One third of the flasks were elicited with MeJA 0.5 mM in the same conditions and the medium was supplemented with 50 mM methyl-β-cyclodextrins (Wacker, Munich, Germany). Stilbene content in the extracellular medium was measured on three flasks at 0, 7, 9, 11, 14, and 16 days after elicitation (except 0.5 mM MeJA one flask and days 0, 7, 9, 11, 14).

### 3.4. Combined Treatment of Methyl-β-Cyclodextrins and Different Elicitors

Seven-day-old flask cell suspensions were diluted in Gamborg medium supplemented with 50 mM methyl-β-cyclodextrins to obtain fresh cultures at 5.6 g DW/L (150 g/L FW). Three days after inoculation, cells were treated with eight elicitors selected from literature: Chitosan 0.1 g/L, laminarin 1 g/L, hydrogen peroxide 5 and 10 mM, jasmonic acid 0.5 and 0.7 mM, sodium orthovanadate 0.5 and 0.7 mM, salicylic acid 0.5 and 0.7 mM, ethephon 0.5 and 0.7 mM, and MeJA 0.5 and 0.7 mM. Controls with acetic acid 0.1% and ethanol were done, as these solvents were used as carrier. Each treatment and control was realized on duplicate flasks. After 10 days of culture, *trans*-resveratrol, ε-viniferin, and δ-viniferin, were quantified in the extracellular medium.

### 3.5. Stirred Bioreactor Cell Culture

The cells were grown either in a 5 L or a 30 L bioreactor with 20 L working volume. The 5 L bioreactor was equipped with a 3-blade marine segment impeller and ring sparger (Biostat Bplus, Sartorius). Vessel geometry was H/D 2:1. The agitation rate was 100 rpm and aeration rate was 0.15 vvm. The bioreactor was inoculated with 300 mL of a 7-day-old cell suspension at approximately 15 g DW/L into 1.2 L of fresh Gamborg medium. The determination of the dry weight was performed after separation of the biomass from the growth medium (10 mL of cell suspension) by centrifugation (5000× *g*; 20 min). The pellet was dried and weighed in an infrared moisture analyzer (MA 150, Sartorius, Göttingen, Germany). Three days after inoculation, the medium was fed at 32.9 g/h until the final volume (4.84 L) was reached 7.3 days later. Elicitation was performed after 11 days of culture at biomass 6.2 g DW/L, by adding an appropriate volume of alcoholic solution of MeJA to obtain a final concentration of 0.7 mM in the bioreactor. After elicitation, stilbenes were quantified in the culture medium on a daily basis.

The 20 L bioreactor (Biostat Cplus, Sartorius) was equipped with baffles and three Rushton 6-blade disk impellers and ring sparger. Vessel geometry was H/D 2:1. The agitation rate was 50 rpm and aeration rate 0.15 vvm. The bioreactor was inoculated with 1.2 L of a 7-day-old cell suspension at approximately 15 g DW/L into 5.0 L fresh Gamborg medium. For experiments with methyl β-cyclodextrins, the Gamborg medium was supplemented with 50 mM of methyl- β-cyclodextrins W7M (Wacker, Munich, Germany). Four days after inoculation, fresh medium was fed at 94 mL/h until the final volume (19.2 L) was reached 9.8 days after inoculation. Elicitation was carried out 11 days after inoculation at 6 g/L DW by adding an alcoholic solution of MeJA for obtaining a final concentration 0.5 mM. After elicitation, stilbenes were quantified in the extracellular medium on a daily basis, except for the experiment conducted with cyclodextrins, for which stilbene content was determined from day 0 (as cyclodextrins elicit the production of resveratrol).

### 3.6. Stilbene Extraction, HPLC Quantification and Identification

Samples of culture medium, 5 mL, were contacted three times with 5 mL ethyl acetate to extract stilbenes dissolved un the culture medium. The organic phase was then evaporated to dryness, resuspended in methanol (1 mL), and filtered through a 0.2 µm PTFE membrane (VWR International, Fontenay sous bois, France).

Stilbene extraction from cells was performed by adding 4 mL of methanol to 50 mg of freeze-dried and ground cells. Extraction was conducted overnight, and after centrifugation (4000× *g*; 10 min), the supernatant was filtered through a 0.2 µm PTFE membrane (VWR International, Fontenay sous bois, France).

Stilbenes adsorbed on resin were extracted in order to be included in the calculation of stilbene content per liter of extracellular medium. The nylon bag containing XAD 1600N resin was contacted with ethanol 96° (190 mL) for 2 h under stirring in the dark. Ethanol was evaporated in a rotary evaporator and dry extract was resuspended in HPLC grade methanol (15.6 mL). After filtration on 0.2 µm PTFE syringe filter, the product was quantified as described below.

Analysis of stilbenes was performed by HPLC (Thermofisher Ultimate3000, Waltham, MA, USA) on a 4.6 × 150 mm Acclaim polar Advantage II Thermo Scientific (3 µm) (Waltham, MA, USA) reverse phase C18 column. The injection volume was 5 µL. The separation was performed at a flow rate of 1 mL/min with two mobile phases: A (0.1% v/v aqueous formic acid) and B (0.1% v/v formic acid in acetonitrile). The HPLC separation conditions were as follows: 0–1 min, 30% B; 1–14 min from 30% to 68% B; 14–14.5 min from 68% to 100% B; 14.5–17.5 min 100% B; 17.5–18 min from 100% to 30% B; and 18–21 min 30% B. The detection was performed at 306 and 286 nm with a diode array detector (Dionex, U3000 DAD, Thermo Scientific, Waltham, MA, USA).

Stilbene contents were estimated from calibration curves prepared with standards (Figure 1): *Trans*-resveratrol (Sigma-Aldrich, Saint Louis, MO, USA), pallidol, ε-viniferin and δ-viniferin (Polyphenol Biotech, Villenave-d’Ornon, France). The detected compounds were identified by comparison of their UV spectra and retention times with those of the pure standards. *Trans*-resveratrol, pallidol, and δ-viniferin were also identified by UPLC–MS/MS analysis. Samples were extracted as described above, evaporated to dryness, resuspended in methanol/water (50,50, v/v), and filtered through a PTFE filter 0.45 µm. The chromatography apparatus from Agilent Technologies (Santa Clara, CA, USA) was coupled to an Esquire 3000 Plus ion trap mass spectrometer using an ESI source (Bruker Daltonics, Billerica, MA, USA). The separation was achieved at a flow rate of 0.4 mL/min with two mobile phases: A (0.1% v/v aqueous formic acid) and B (0.1% v/v formic acid in acetonitrile) on an Agilent C18 column (2.1 × 100 mm, 1.8 µm). The injection volume was 1 µL. The UPLC separation conditions were as follows: 0–0.4 min 17% B; 0.4–4.4 min from 17% to 30% B; 4.4–7.4 min from 30% to 38% B; 7.4–9 min from 38% to 50% B; 9–10 min from 50% to 100% B; 10–11 min 100% B; 11–11.2 min from 100% to 17% B; 11.2–14.2 min 17% B. The chromatogram was monitored at 286 nm. After DAD detector, the UPLC output was split in the MS detector. Analysis was performed in positive mode with a range of *m/z* 100–1400 with capillary voltage 4.6 kV, nebulizer pressure of 40 psi at 365 °C and drying gas (nitrogen) at 10 L/min. Data analysis was performed with Hystar 3.0 software.

## 4. Conclusions

The goal of this study was to increase the knowledge on the bioproduction of resveratrol oligomers in grapevine cell suspension (*V. labrusca*). Three resveratrol dimers were identified in the cell suspension: pallidol, δ-viniferin, and ɛ-viniferin. Optimization of elicitation by MeJA alone demonstrated a clear correlation between biomass, MeJA, and sugar concentrations. For the first time, the effect of methyl-β-cyclodextrins and adsorbent resin on resveratrol oligomers production was studied. In their presence, the maximum concentrations reached 6141 mg/L for resveratrol, 900 mg/L for pallidol, 537 mg/L for δ-viniferin, and 500 mg/L for ɛ-viniferin. We provide evidence that methyl-β-cyclodextrins and adsorbent resin combined with a specific elicitor in optimized conditions allowed to modulate stilbene profile for the production of target molecule. The recent discovery of new dimers in *V. labrusca* cell suspension (leachianols F and G, labruscol) opens the production of a larger diversity of resveratrol derivatives [4,5].

Moreover, we succeeded in the scale-up to 20 L culture volume without loss in growth or elicitation efficiency. Finally this work provides new strategies for the bio-production of resveratrol oligomers in a significant amount.

## Figures and Tables

**Table 1:** Statistical analysis by Student’s *t*-test of *trans*-resveratrol yield from full factorial design of experiment. Coefficients are normalized (e.g., corresponding to normalized parameters).

Factor ^a^	Coefficient Estimate	Standard Error	*t*-Student	*p*-Value
Intercept	11.56	0.91	12.6	<0.0001 *
B	−7.41	1.02	−7.3	<0.0001 *
M	5.12	1.02	5.0	<0.0001 *
S	0.20	0.97	0.2	0.84
B × M	−2.84	1.02	−2.8	0.011 *
B × S	4.97	1.02	4.9	<0.0001 *
M × S	−0.77	1.02	−0.8	0.46
B × M × S	2.91	1.02	2.9	0.0095 *

^a^ B, biomass concentration; M, methyl jasmonate concentration; S, sucrose concentration added. * *p* < 0.05 is significant, R^2^ = 0.86.

**Table 2:** Statistical analysis by Student’s *t*-test of pallidol yield from full factorial design of experiment. Coefficients are normalized (e.g., corresponding to normalized parameters).

Factor ^a^	Coefficient Estimate	Standard Error	*t*-Student	*p*-Value
Intercept	11.71	0.46	25.3	<0.0001 *
B	−5.14	0.51	−10.0	<0.0001 *
M	0.50	0.51	1.0	0.34
S	1.75	0.49	3.6	0.0019 *
B × M	0.40	0.51	0.8	0.45
B × S	3.34	0.51	6.5	<0.0001 *
M × S	−2.19	0.51	−4.3	<0.0001 *
B × M × S	1.86	0.51	3.6	0.0017 *

^a^ B, biomass concentration; M, methyl jasmonate concentration; S, sucrose concentration added. * *p* < 0.05 is significant, R^2^ = 0.91.

## Supplementary Materials

The following are available online at https://www.mdpi.com/2223-7747/8/12/567/s1, **Figure S1**: Time course of resveratrol (a) and pallidol (b) accumulation in the *V. labrusca* cell suspension extracellular medium (100 mL culture volume in flask) after elicitation with 0.5 or 0.7 mM methyl jasmonate and 5.6 or 11.2 g DW/L biomass, **Figure S2**: Comparison of ten adsorbent resins from Dow chemicals (XAD7HP, FPX68, XAD1600N, OptiporeSD2, XAD4 and FPX66) and Diaion (HP20, SP207, SP70 and SP850). 500 mg of each resin was put in contact with 50 mL extracellular medium from cell suspension containing stilbenes, at different concentration, for 2 hours. At the end of experiment stilbenes were quantified in the medium. Adsorption isotherm curves were determined for each resin and each stilbene (adsorbed amount vs. concentration in liquid phase). Then Qmax (adsorption capacity) and Kaffinity (affinity coefficients) were estimated to fit experimental points with a competitive Langmuir model (equation right below). Affinity coefficients are presented for resveratrol (Kaff RV), pallidol (Kaff Pal) and for ε- and δ-viniferins gathered together (Kaff others) and for all stilbenes combined (Kaff global). XAD1600N revealed a great affinity for all compounds. Further experiments demonstrated that all stilbenes can be recovered efficiently (> 95%) by desorption in ethanol (ratio > 100 mL/g resin).

## References

[1] Pawlus A.D., Waffo-Téguo P., Shaver J., Mérillon J.-M. (2012). Stilbenoid chemistry from wine and the genus *Vitis*, a review. Oeno One.

[2] Anekonda T.S. (2006). Resveratrol-a boon for treating Alzheimer’s disease?. Brain. Res. Rev..

[3] Baur J.A., Sinclair D.A. (2006). Therapeutic potential of resveratrol: The *in vivo* evidence. Nat. Rev. Drug Discov..

[4] Nivelle L., Hubert J., Courot E., Jeandet P., Aziz A., Nuzillard J.-M., Renault J.-H., Clément C., Martiny L., Delmas D. (2017). Anti-cancer activity of resveratrol and derivatives produced by grapevine cell suspensions in a 14 L stirred bioreactor. Molecules.

[5] Nivelle L., Hubert J., Courot E., Borie N., Renault J.H., Nuzillard J.M., Harakat D., Clément C., Martiny L., Delmas D. (2017). Characterization of labruscol, a new resveratrol dimer produced by grapevine cell suspensions on Human Skin Melanoma Cancer Cell Line HT-144. Molecules.

[6] He S., Yan X. (2013). From resveratrol to its derivatives: New sources of natural antioxidant. Curr. Med. Chem..

[7] Ohyama M., Tanaka T., Ito T., Iinuma M., Bastow K.F., Lee K.-H. (1999). Antitumor agents 200. Cytotoxicity of naturally occurring resveratrol oligomers and their acetate derivatives. Bioorg. Med. Chem. Lett..

[8] Yim N., Ha D.T., Trung T.N., Kim J.P., Lee S., Na M., Jung H., Kim H.S., Kim Y.H., Bae K. (2010). The antimicrobial activity of compounds from the leaf and stem of *Vitis amurensis* against two oral pathogens. Bioorg. Med. Chem. Lett..

[9] Colin D., Lancon A., Delmas D., Lizard G., Abrossinow J., Kahn E., Jannin B., Latruffe N. (2008). Antiproliferative activities of resveratrol and related compounds in human hepatocyte derived HepG2 cells are associated with biochemical cell disturbance revealed by fluorescence analyses. Biochimie.

[10] Kim H.J., Chang E.J., Bae S.J., Shim S.M., Park H.D., Rhee C.H., Park J.H., Choi S.W. (2002). Cytotoxic and antimutagenic stilbenes from seeds of *Paeonia lactiflora*. Arch. Pharm. Res..

[11] Richard T., Poupard P., Nassra M., Papastamoulis Y., Iglésias M.-L., Krisa S., Waffo-Teguo P., Mérillon J.-M., Monti J.-P. (2011). Protective effect of ε-viniferin on β-amyloid peptide aggregation investigated by electrospray ionization mass spectrometry. Bioorg. Med. Chem..

[12] Zghonda N., Yoshida S., Ezaki S., Otake Y., Murakami C., Mliki A., Ghorbel A., Miyazaki H. (2012). ε-Viniferin is more effective than its monomer resveratrol in improving the functions of vascular endothelial cells and the heart. Biosci. Biotechnol. Biochem..

[13] Donnez D., Jeandet P., Clément C., Courot E. (2009). Bioproduction of resveratrol and stilbene derivatives by plant cells and microorganisms. Trends Biotechnol..

[14] Lambert C., Richard T., Renouf E., Bisson J., Waffo-Téguo P., Bordenave L., Ollat N., Mérillon J.-M., Cluzet S. (2013). Comparative analyses of stilbenoids in canes of major *Vitis vinifera* L. cultivars. J. Agric. Food Chem..

[15] Pawlus A.D., Sahli R., Bisson J., Rivière C., Delaunay J.-C., Richard T., Gomès E., Bordenave L., Waffo-Téguo P., Mérillon J.-M. (2013). Stilbenoid profiles of canes from *Vitis* and *Muscadinia* species. J. Agric. Food Chem..

[16] Eibl R., Meier P., Stutz I., Schildberger D., Huhn T., Eibl D. (2018). Plant cell culture technology in the cosmetics and food industries current state and future trends. Appl. Microbiol. Biotechnol..

[17] Melchior F., Hohmann F., Schwer B., Kindl H. (1991). Induction of stilbene synthase by *Botrytis cinerea* in cultured grapevine cells. Planta.

[18] Jeandet P., Clément C., Courot E. (2014). Resveratrol production at large scale using plant cell suspensions. Eng. Life Sci..

[19] Donnez D., Kim K.-H., Antoine S., Conreux A., De Luca V., Jeandet P., Clément C., Courot E. (2011). Bioproduction of resveratrol and viniferins by an elicited grapevine cell culture in a 2L stirred bioreactor. Process Biochem..

[20] Krisa S., Larronde F., Budzinski H., Decendit A., Deffieux G., Mérillon J.-M. (1999). Stilbene production by *Vitis vinifera* cell suspension cultures: Methyl jasmonate induction and ^13^C Biolabeling. J. Nat. Prod..

[21] Vitrac X., Krisa S., Decendit A., Vercauteren J., Nuhrich A., Monti J.-P., Deffieux G., Mérillon J.-M. (2002). Carbon14 biolabelling of wine polyphenols in *Vitis vinifera* cell suspension cultures. J. Biotechnol..

[22] Belchí-Navarro S., Almagro L., Lijavetzky D., Bru R., Pedreño M.A. (2012). Enhanced extracellular production of *trans*-resveratrol in *Vitis vinifera* suspension cultured cells by using cyclodextrins and methyljasmonate. Plant Cell Rep..

[23] Lijavetzky D., Almagro L., Belchi-Navarro S., Martínez-Zapater J.M., Bru R., Pedreño M.A. (2008). Synergistic effect of methyljasmonate and cyclodextrin on stilbene biosynthesis pathway gene expression and resveratrol production in Monastrell grapevine cell cultures. BMC Res. Notes.

[24] Belchí-Navarro S., Abellan R.M., Pedreño M.A., Almagro L. (2019). Production and localization of hydrogen peroxide and nitric oxide in grapevine cells elicited with cyclodextrins and methyl jasmonate. J. Plant Physiol..

[25] Vuong T.V., Franco C., Zhang W. (2014). Treatment strategies for high resveratrol induction in *Vitis vinifera* L. cell suspension culture. Biotechnol. Rep..

[26] Yue X., Zhang W., Deng M. (2011). Hyper-production of 13C-labeled *trans*-resveratrol in *Vitis vinifera* suspension cell culture by elicitation and in situ adsorption. Biochem. Eng. J..

[27] Lucas-Abellán C., Fortea I., López-Nicolás J.M., Núñez-Delicado E. (2007). Cyclodextrins as resveratrol carrier system. Food Chem..

[28] Larronde F., Richard T., Delaunay J.-C., Decendit A., Monti J.-P., Krisa S., Mérillon J.M. (2005). New stilbenoid glucosides isolated from *Vitis vinifera* cell suspension cultures (cv. Cabernet Sauvignon). Planta Med..

[29] Mérillon J.M., Fauconneau B., Waffo-Téguo P., Barrier L., Vercauteren J., Huguet F. (1997). Antioxidant activity of the stilbene astringin, newly extracted from *Vitis vinifera* cell cultures. Clin. Chem..

[30] Mulinacci N., Innocenti M., Santamaria A.R., La Marca G., Pasqua G. (2010). High-performance liquid chromatography/electrospray ionization tandem mass spectrometric investigation of stilbenoids in cell cultures of *Vitis vinifera* L., cv. Malvasia. Rapid Commun. Mass Spectrom..

[31] Santamaria A.R., Antonacci D., Caruso G., Cavaliere C., Gubbiotti R., Laganà A., Valletta A., Pasqua G. (2010). Stilbene production in cell cultures of *Vitis vinifera* L. cvs Red Globe and Michele Palieri elicited by methyl jasmonate. Nat. Prod. Res..

[32] Waffo-Téguo P., Hawthorne M.E., Cuendet M., Mérillon J.M., Kinghorn A.D., Pezzuto J.M., Mehta R.G. (2001). Potential cancer-chemopreventive activities of wine stilbenoids and flavans extracted from grape (*Vitis vinifera*) cell cultures. Nutr. Cancer.

[33] Waffo-Téguo P., Fauconneau B., Deffieux G., Huguet F., Vercauteren J., Mérillon J.M. (1998). Isolation, identification, and antioxidant activity of three stilbene glucosides newly extracted from *Vitis vinifera* cell cultures. J. Nat. Prod..

[34] Taurino M., Ingrosso I., D’amico L., De Domenico S., Nicoletti I., Corradini D., Santino A., Giovinazzo G. (2015). Jasmonates elicit different sets of stilbenes in *Vitis vinifera* cv. Negramaro cell cultures. SpringerPlus.

[35] Chastang T., Pozzobon V., Taidi B., Courot E., Clément C., Pareau D. (2018). Resveratrol production by grapevine cells in fed-batch bioreactor: Experiments and modelling. Bioch. Eng. J..

[36] Belhadj A., Telef N., Saigne C., Cluzet S., Barrieu F., Hamdi S., Mérillon J.M. (2008). Effect of methyl jasmonate in combination with carbohydrates on gene expression of PR proteins, stilbene and anthocyanin accumulation in grapevine cell cultures. Plant Physiol. Biochem..

[37] Ferri M., Righetti L., Tassoni A. (2011). Increasing sucrose concentrations promote phenylpropanoid biosynthesis in grapevine cell cultures. J. Plant Physiol..

[38] Cruz E.F., Cerezo A.B., Cantos-Villar E., Richard E., Troncoso A.M., Garcia-Parilla M.C. (2019). Inhibition of VEGFR-2 phosphorylation and effects on downstream signaling pathways in cultivated human endothelial cells by stilbenes from *Vitis* spp.. J. Agric. Food Chem..

[39] Li C., Xu X., Tao Z., Wang X.J., Pan Y. (2015). Resveratrol dimers, nutritional components in grape wine, are selective ROS scavengers and weak Nrf2 activators. Food Chem..

[40] Morales M., Bru R., García-Carmona F., Barceló A.R., Pedreño M.A. (1998). Effect of dimethyl-β-cyclodextrins on resveratrol metabolism in Gamay grapevine cell cultures before and after inoculation with shape *Xylophilus ampelinus*. Plant Cell Tissue Organ Cult..

[41] Oliva E., Mathiron D., Bertaut E., Landy D., Cailleu D., Pilard S., Clément C., Courot E., Bonnet V., Djedaini-Pilard F. (2018). Physico-chemical studies of resveratrol, methyl-jasmonate and cyclodextrins interactions: An approach to resveratrol bioproduction optimization. RSC Adv..

[42] Chang X., Heene E., Qiao F., Nick P. (2011). The Phytoalexin Resveratrol Regulates the Initiation of hypersensitive cell death in *Vitis* cell. PLoS ONE.

[43] Bru R., Sellés S., Casado-Vela J., Belchí-Navarro S., Pedreño M.A. (2006). Modified cyclodextrins are chemically defined glucan inducers of defense responses in grapevine cell cultures. J. Agric. Food Chem..

[44] Santamaria A.R., Mulinacci N., Valetta A., Innocenti M., Pasqua G. (2011). Effects of elicitors on the production of resveratrol and viniferins in cell culture of *Vitis vinfera* L. cv Italia. J. Agric. Food Chem..

[45] Gabaston J., Leborgne C., Valls J., Renouf E., Richard T., Waffo-Teguo P., Mérillon J.-M. (2018). Subcritical water extraction of stilbenes from grapevine by-products: A new green chemistry approach. Ind. Crop. Prod..

[46] Barceló A.R., Pomar F., López-Serrano M., Pedreño M.A. (2003). Peroxidase: A multifunctional enzyme in grapevines. Funct. Plant Biol..

[47] Vera-Urbina J.C., Sellès-Marchart S., Martinez-Esteso M.J., Pedreno M.A., Bru-Martinez R., Delmas D. (2013). Production of grapevine cell biomass (*Vitis vinifera* L. Gamay) and resveratrol in custom and commercial bioreactors using cyclodextrins and methyl jasmonate elicitors. Resveratrol: Source, Production, and Health Benefits.

[48] Bru M.R., Pedreno G.M. (2007). Method for the Production of Resveratrol in Cell Cultures. U.S. Patent.

[49] Chen J., Hall D.E., Murata J., De Luca V. (2006). L-Alanine induces programmed cell death in *V. labrusca* cell suspension cultures. Plant Sci..

[50] Gamborg O.L., Miller R.A., Ojima K. (1968). Plant cell cultures 1. Nutrients requirements of suspension cultures of soybean root cells. Exp. Cell. Res..

