# Supplementary material for: Optimize, Modulate, and Scale-up Resveratrol and Resveratrol Dimers Bioproduction in Vitis labrusca L. Cell Suspension from Flasks to 20 L Bioreactor
Source: Plants (Basel). 2019 Dec 4;8(12):567. doi: 10.3390/plants8120567 (PMC6963533; doi:10.3390/plants8120567)
Supplement: Supplementary file 1 [file plants-08-00567-s001.pdf]

Supplementary Materials:

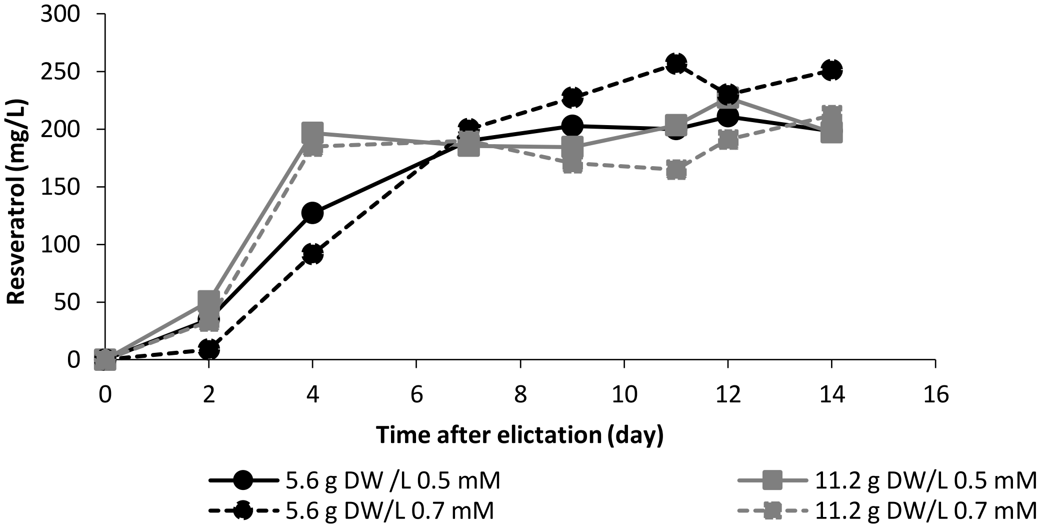

(a)

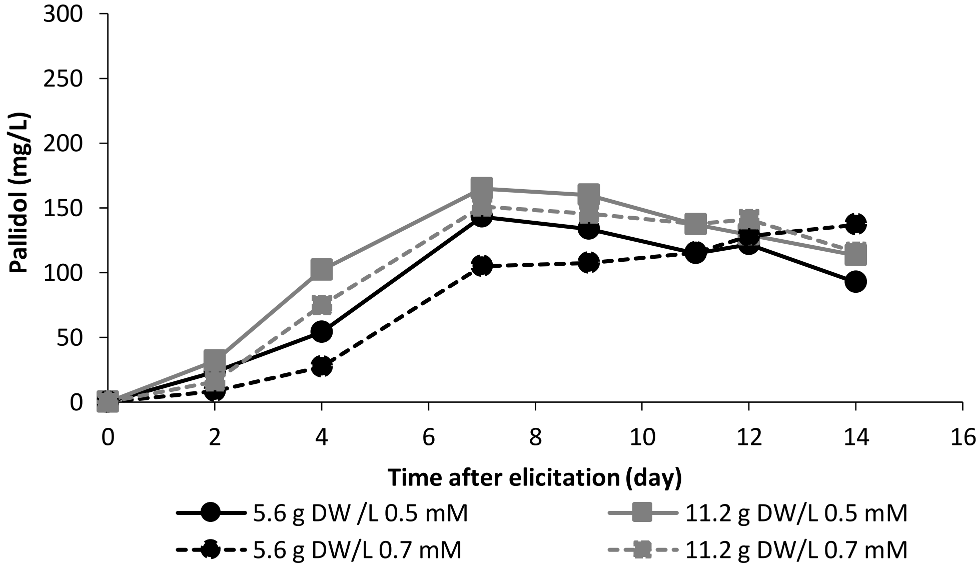

(b)

Figure S1. Time course of resveratrol (**a**) and pallidol (**b**) accumulation in the *V. labrusca* cell suspension extracellular medium (100 mL culture volume in flask) after elicitation with 0.5 or 0.7 mM methyl jasmonate and 5.6 or 11.2 g DW/L biomass.

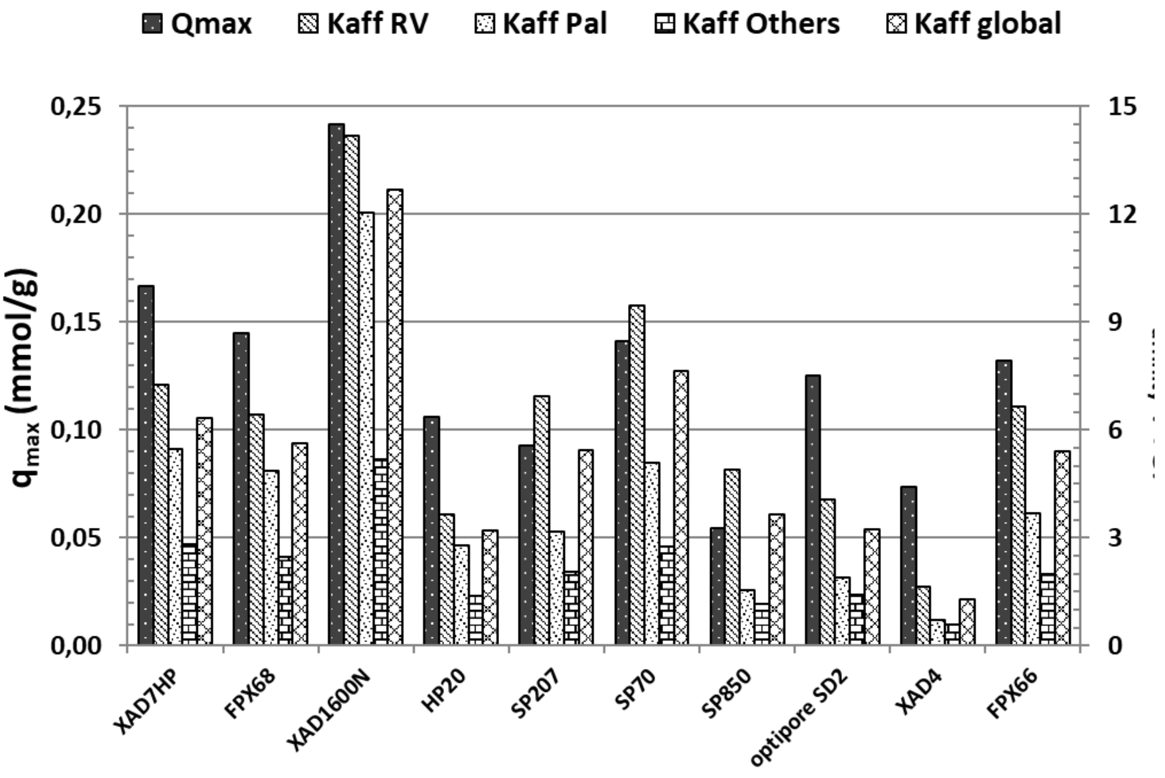

(a)

Figure S2. Comparison of ten adsorbent resins from Dow chemicals (XAD7HP, FPX68, XAD1600N, OptiporeSD2, XAD4 and FPX66) and Diaion (HP20, SP207, SP70 and SP850). 500 mg of each resin was put in contact with 50 mL extracellular medium from cell suspension containing stilbenes, at different concentration, for 2 hours. At the end of experiment stilbenes were quantified in the medium. Adsorption isotherm curves were determined for each resin and each stilbene (adsorbed amount vs. concentration in liquid phase). Then Qmax (adsorption capacity) and Kaffinity (affinity coefficients) were estimated to fit experimental points with a competitive Langmuir model (equation right below). Affinity coefficients are presented for resveratrol (Kaff RV), pallidol (Kaff Pal) and for ε- and δ-viniferins gathered together (Kaff others) and for all stilbenes combined (Kaff global). XAD1600N revealed a great affinity for all compounds. Further experiments demonstrated that all stilbenes can be recovered efficiently (> 95%) by desorption in ethanol (ratio > 100 mL/g resin).

$$\boldsymbol{q_i} = \frac{\boldsymbol{Q_{max} K_{aff_i} C_i}}{\boldsymbol{Q_{max} + \sum_j K_{aff_j} C_j}}$$

where $\boldsymbol{q_i}$ corresponds to stilbene **i** adsorbed amount on resin (in mmol/g);

$\boldsymbol{C_i}$ and $\boldsymbol{C_j}$ correspond respectively to stilbene **i** and **j** concentration in liquid phase (in mmol/L);

$\boldsymbol{Q_{max}}$ corresponds to resin adsorption capacity (in mmol/g);

$\boldsymbol{K_{aff_i}}$ and $\boldsymbol{K_{aff_j}}$ correspond, respectively, to stilbene **i** and **j** affinity coefficient (in L/g).
